# Effect of Methyl Gallate on Physiological and Gut Microbiota-Associated Responses Following a Single Intramuscular Administration of Tylosin in Weaned Piglets

**DOI:** 10.3390/pathogens15070778

**Published:** 2026-07-22

**Authors:** Hae-Yeon Cho, Syed Al Jawad Sayem, Ga-Yeong Lee, Jonghyun Park, Eon-Bee Lee, Mi-Hyang Hwangbo, Seung-Chun Park

**Affiliations:** 1 Laboratory of Veterinary Pharmacology, Institute for Veterinary Biomedical Science, College of Veterinary Medicine, Kyungpook National University, Daegu 41566, Republic of Korea; whgodus@knu.ac.kr (H.-Y.C.); aljawadsayem@knu.ac.kr (S.A.J.S.); yeong1129@knu.ac.kr (G.-Y.L.); 2 DIVA Bio Incorporation, Iksan 54531, Republic of Korea; accumedix@accumedix.co.kr; 3 Department of Aquatic Life Medicine, Pukyong National University, Busan 48513, Republic of Korea; eonbee@pknu.ac.kr; 4 Department of Food and Nutrition, Keimyung College University, Daegu 42601, Republic of Korea; mhhwangbo@kmcu.ac.kr

**Keywords:** tylosin, methyl gallate, gut microbiota, weaned piglets, immune modulation, *Lactobacillus* spp.

## Abstract

Tylosin (TYL), a macrolide antibiotic, is commonly used to treat respiratory infections in weaned piglets. Despite its utility, TYL administration is associated with adverse side effects, including immune dysregulation and gut microbiota imbalance. This study evaluated whether methyl gallate (MG), a natural polyphenolic compound with antimicrobial and immunomodulatory properties, could mitigate these side effects of TYL. Forty weaned piglets were randomly assigned to a saline control group, a TYL (10 mg/kg) group, an MG (10 mg/kg) group, and a TYL + MG (10 mg/kg each) group. Changes in body weight, hematological parameters, plasma cytokines, culturable fecal bacterial populations, and 16S rRNA-based gut microbiota composition were evaluated in all weaned piglets. There were no significant differences (*p* > 0.05) in ADG, ADFI, or FE among the treatment groups. Hematological analysis revealed significant changes in neutrophil and eosinophil counts, showing significant fluctuations in the MG-treated group compared with the control. TYL administration significantly decreased TNF-α and IL-1β levels while increasing IL-10 and IL-1ra concentrations, indicating immunomodulatory effects. In contrast, MG significantly increased adaptive immune-associated cytokines, including IL-2, IL-4, IL-6, and IL-12. Culture-based fecal microbiological analysis revealed that TYL reduced *Lactobacillus* abundance and lowered the culture-based *Lactobacillus*/*Enterobacteriaceae* ratio, suggesting acute dysbiosis. Co-administration of MG partially attenuated these microbiota-associated alterations and promoted the recovery of bacterial populations. Moreover, 16S rRNA sequencing demonstrated that similar relative abundances of Proteobacteria were observed in the TYL and TYL + MG groups. Therefore, no clear reduction in relative abundance of Proteobacteria was demonstrated under the present experimental conditions. These findings support the possibility that MG may partially attenuate selected TYL-associated immunological and gut microbiota-related changes under the conditions of the present exploratory study. A graphical summary of the study design and the principal findings is presented in the Graphical Abstract.

## 1. Introduction

International regulatory bodies are currently advocating reduced antibiotic use within the One Health framework to combat the rapid emergence of antibiotic-resistant bacteria [1,2,3,4]. Tylosin (TYL) is a widely used macrolide antibiotic frequently administered to weaned piglets to manage respiratory infections [5,6]. However, conventional administration via feed or water has been linked to the development of resistant strains and significant gut microbiota dysbiosis. Aarestrup et al. [7] further demonstrated that oral TYL administration directly contributes to antibiotic resistance in both intestinal and skin microbiota. To mitigate these risks, intramuscular (IM) injection is often considered an alternative to reduce total antibiotic exposure. Nevertheless, TYL exhibits a large volume of distribution and extensive biliary excretion. As a result, IM administration still leads to intestinal exposure via enterohepatic circulation, which can cause temporary microbial imbalances in pigs and dogs [8,9].

The weaning period is a critical developmental stage with sudden changes in diet and environmental stress, making piglets particularly susceptible to enteric infections and gut microbiota disorders [10]. During this period, the intestinal microbiota plays a pivotal role in maintaining immune homeostasis and preserving the epithelial barrier integrity. However, antibiotic treatment, including TYL administration, can disrupt the intestinal microbiota, resulting in an increased relative abundance of Proteobacteria and a reduction in beneficial culture-based Lactobacillus populations. Such alterations may reduce microbial diversity, promote microbiota-associated dysbiosis, and impair host immune regulation, ultimately compromising the therapeutic outcome of antibiotic treatment [3,11,12]. Accordingly, adjunctive therapeutic strategies capable of minimizing antibiotic-associated microbiota disturbances while preserving antimicrobial efficacy are needed in weaned piglets.

Polyphenolic compounds possess a wide range of biological activities, including antioxidant, anti-inflammatory, antimicrobial, and gut microbiota-modulating properties [13,14,15]. Among these compounds, methyl gallate (MG), a naturally occurring gallotannin ester, exerts diverse pharmacological effects through modulation of the AMPK/NF-κB and TLR4/NF-κB signaling pathways [15]. MG inhibits the adhesion and invasion of *Salmonella* Typhimurium by downregulating virulence- and quorum-sensing-related genes, including *sdiA, sipB,* and *cheY* [16,17]. Moreover, when combined with TYL, MG disrupts bacterial membrane integrity and reduces the intracellular survival of *Salmonella enterica* subsp. *enterica* serovar Typhimurium [16,18]. MG has also been reported to protect intestinal barrier function through modulation of the NF-κB and MAPK pathways [15,19,20]. Consistent with these findings, other polyphenols, including apple peel and tea polyphenols, have been shown to restore microbial richness and beneficial bacterial populations following antibiotic-induced dysbiosis [21,22]. Collectively, these observations suggest that MG may have the potential to attenuate TYL-associated microbiota disturbances.

Despite these promising findings, evidence regarding the effects of intramuscular TYL administration on immune responses and gut microbiota composition in weaned piglets remains limited. Furthermore, it remains unclear whether co-administration of MG can influence TYL-associated physiological, immune-associated, and microbiota-associated responses. Therefore, this exploratory study investigated whether co-administration of MG influences selected physiological, immunological, and microbiota-associated responses following IM administration of TYL in weaned piglets.

## 2. Materials and Methods

### 2.1. Animal Experimental Design

**Figure 1:**
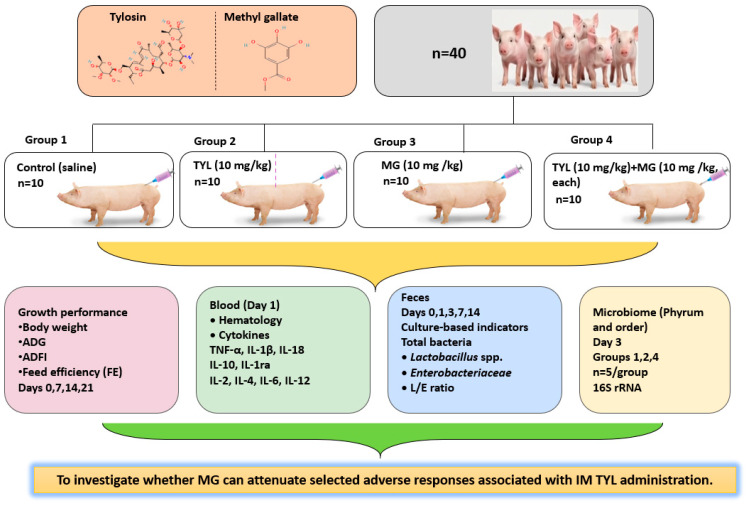
Experimental design and treatment groups. All doses were administered once on day 0, based on body weight. Abbreviations: BW, body weight; MG, methyl gallate; TYL, tylosin; IM, intramuscular.

A total of forty healthy crossbred piglets (Yorkshire × Landrace) were 28 days old at weaning and underwent a 7-day acclimation period, meaning the treatment was administered at 5 weeks of age. The initial body weight (BW) of the animals ranged from 8.2 to 12.7 kg. Weaned piglets were randomly allocated into four treatment groups (*n* = 10 per group): Group 1 (sterile 0.9% saline; vehicle control), Group 2 (TYL, 10 mg/kg), Group 3 (MG, 10 mg/kg), and Group 4 (TYL + MG, 10 mg/kg each) (Figure 1). TYL (50 mg/mL, TYL base), MG (50 mg/mL) or TYL + MG (100 mg/mL) injectable solution was prepared using 50% propylene glycol with 4% benzyl alcohol as a vehicle. Injectable TYL base was provided by Samyang Anipharm (Seoul, Korea). All treatments were given as a single IM administration on day 0, and the dosage was calculated based on each piglet’s body weight. The piglets were kept in a controlled environment with a regular light–dark cycle. To prevent heat stress and keep the conditions consistent, we adjusted the temperature according to their growth stage: 28–30 °C for the early weaning period (weeks 1–2) and 23–25 °C for the late weaning period (from week 3 onwards). Each treatment group was assigned to a separate pen (*n* = 10 per pen) equipped with plastic-coated expanded flooring. All piglets were provided the same basal diet formulated to contain 20.47% crude protein (CP, as-fed basis), with standardized ileal digestible amino acids balanced using crystalline supplements; thus, dietary CP content remained constant across treatments (Table 1).

### 2.2. Growth Performance and Hematological Assessment

Individual body weight (BW) was recorded on days 0, 7, 14, and 21 post-treatment. Average daily gain (ADG) was calculated based on changes in BW over each interval. Average daily feed intake (ADFI) was determined at the pen level based on residual feed measurements. Therefore, feed intake and feed efficiency should be interpreted as pen-level observations. Feed efficiency (FE) was calculated as the ratio of ADG to ADFI.

Blood samples (1 mL) were collected via jugular venipuncture into EDTA-coated tubes one day post-administration for hematological evaluation. Complete blood counts (CBC) were analyzed using a Hemavet 950FS analyzer (Drew Scientific, Oxford, UK). The parameters assessed included leukocyte profiles (white blood cells [WBC], neutrophils, lymphocytes, monocytes, eosinophils, and basophils), erythrocyte indices (red blood cells [RBC], hemoglobin [Hb], hematocrit [HCT], mean corpuscular volume [MCV], mean corpuscular hemoglobin [MCH], mean corpuscular hemoglobin concentration [MCHC], and red cell distribution width [RDW]), and platelet indices (platelet count [PLT] and mean platelet volume [MPV]).

### 2.3. Plasma Cytokine Analysis

This study evaluated acute physiological responses after a single IM administration on early plasma cytokine-microbiota changes using a multiplex bead-based immunoassay (Luminex system; LABIS KOMA, Seoul, Republic of Korea). The cytokine panel included tumor necrosis factor-α (TNF-α), interleukin-1α (IL-1α), interleukin-1β (IL-1β), interleukin-18 (IL-18), interleukin-10 (IL-10), interleukin-1 receptor antagonist (IL-1ra), interleukin-4 (IL-4), interleukin-6 (IL-6), interleukin-2 (IL-2), and interleukin-12 (IL-12), providing a comprehensive immunological profile.

### 2.4. Culturable Fecal Microbiota Analysis

To evaluate changes in fecal microbial composition, culture-based microbial indicators were enumerated using selective media. Piglets received IM injections of TYL alone (Group 2), MG alone (Group 3), or a combination of TYL and MG (Group 4). Fecal microorganisms were collected and cultured on days 1, 3, 7, and 14 after injection using different selective media. Total aerobic bacteria were enumerated on Plate Count Agar (PCA), while total anaerobic bacteria were cultured on Blood Agar (BA) at 35 °C for 48 h under anaerobic conditions. Lactic acid bacteria were grown on de Man, Rogosa, and Sharpe (MRS) agar, and Bifidobacterium spp. were selectively isolated using Transgalactosylated Oligosaccharides (TOS-MUP) agar supplemented with mupirocin, both maintained under anaerobic conditions. For the identification of enteric populations, Gram-negative bacteria, including *Escherichia coli*, were cultured on MacConkey (MAC) agar. Staphylococci were differentiated on Mannitol Salt (MS) agar based on their mannitol fermentation profile. Additionally, Clostridium species, such as *Clostridium perfringens*, were isolated using Tryptose Sulfite Neomycin (TSN) agar under strict anaerobic conditions. All plates were incubated at 35–37 °C for 24 to 48 h. Following incubation, the resulting colonies were counted, and bacterial concentrations were expressed as log10 colony-forming units per gram (log10 CFU/g) of fecal sample.

### 2.5. DNA Extraction, 16S rRNA Sequencing, and Microbiota Analysis

Fecal samples were collected on day 3 from the experimental piglets (Groups 1, 2, and 4; *n* = 5 per group). This time point was specifically selected based on preliminary culture-based microbiota data, which indicated the most significant divergence in the *Lactobacillus* to *E. coli* ratio among the groups. Following collection, the samples were immediately snap-frozen and stored at −80 °C until further analysis. Total genomic DNA was extracted from the fecal samples using the Ultra-Clean Fecal DNA Isolation Kit (MoBio Laboratories, Carlsbad, CA, USA) according to the manufacturer’s instructions. The quantity and quality of the extracted DNA were assessed using a spectrophotometer (e.g., NanoDrop ND-1000, Thermo Fisher Scientific, Waltham, MA, USA). The extracted DNA samples were submitted to CJ Bioscience, Inc. (Seoul, South Korea) for 16S rRNA gene amplicon sequencing. The hypervariable V3–V4 regions of the bacterial 16S rRNA gene were amplified using specific primers (341F and 805R). Following library preparation, paired-end sequencing was performed on the Illumina MiSeq platform (Illumina, San Diego, CA, USA). The raw sequencing data were processed using the proprietary EzBioCloud microbiota taxonomic profiling pipeline (CJ Bioscience, Inc., Seoul, Republic of Korea). The workflow included quality filtering and the removal of chimeric sequences. Taxonomic assignment was performed against the EzBioCloud database, and operational taxonomic units (OTUs) were defined by clustering sequences at a 97% similarity threshold to ensure robust comparative analysis. To evaluate the microbial community shifts induced by the TYL and TYL + MG treatments, alpha diversity indices, including the Shannon and Chao1 indices, were calculated to assess microbial richness and evenness. Furthermore, beta diversity was evaluated via Principal Coordinate Analysis (PCoA) based on UniFrac distances to compare the microbial community structures across the treatment groups.

### 2.6. Statistical Analysis

All statistical analyses were conducted using SAS 9.4 (SAS Institute Inc., Cary, NC, USA) and R version 4.1.4 (R Foundation for Statistical Computing, Vienna, Austria). Prior to the main analyses, data distribution and homogeneity of variance were assessed using the Shapiro–Wilk and Levene’s tests, respectively. Growth performance, hematological, and cytokine data were analyzed using one-way analysis of variance (ANOVA). Tukey’s honestly significant difference (HSD) test was applied for post hoc comparisons between treatment groups. Repeated culture-based microbiological measurements collected over time were analyzed using repeated-measures ANOVA to evaluate treatment, sampling day, and treatment × day interaction effects. For the 16S rRNA gut microbiota data, an exploratory analysis was performed. Alpha diversity indices were evaluated using either one-way ANOVA or the Kruskal–Wallis test, depending on data normality. Beta diversity was assessed using permutational multivariate analysis of variance (PERMANOVA). All statistical tests were two-tailed, and statistical significance was set at *p* < 0.05.

## 3. Results

### 3.1. Growth Performance

To evaluate the effects of TYL alone (Group 2), MG alone (Group 3), and TYL + MG combination (Group 4) on growth performance, average daily gain (ADG), average daily feed intake (ADFI) and feed efficiency (FE) were measured over a 21-day period after treatments following acclimatization.

**Figure 2:**
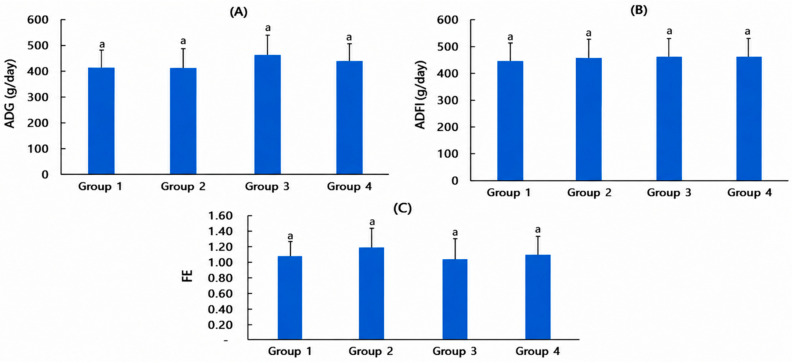
Growth performance of TYL, methyl gallate, and their combination in weaned piglets. Piglets were divided into four groups: saline control (Group 1), TYL alone (Group 2, 10 mg/kg IM), MG alone (Group 3, 10 mg/kg IM), and TYL + MG (Group 4, each 10 mg/kg IM). (**A**) Average daily gain (ADG, g/day), (**B**) average daily feed intake (ADFI, g/day), and (**C**) feed efficiency (FE). The same lowercase superscript letter ‘a’ above the bars indicates that no statistically significant differences were observed among the experimental groups (*p* > 0.05).

The ADG values are presented in Figure 2A. The ADG values were 414.29 ± 52.16 g/day in Group 1, 411.43 ± 61.00 g/day in Group 2, 467.86 ± 112.65 g/day in Group 3, and 442.14 ± 85.94 g/day in Group 4. Group 3 tended to have higher mean ADG values than the other groups; however, the difference was not statistically significant (*p* = 0.378). The ADFI values are illustrated in Figure 2B. The ADFI values for Groups 1 to 4 were 444.29 ± 80.70 g/day, 457.86 ± 73.05 g/day, 462.86 ± 42.91 g/day and 467.14 ± 68.43 g/day, respectively. FE values are presented in Figure 2C. Group 2 exhibited a relatively higher FE value (1.054 ± 0.316) (*p* = 0.760). From these results, MG did not adversely affect growth performance, either alone or when co-administered with TYL.

### 3.2. Hematological Indices

To evaluate the physiological effects of IM administration of TYL, MG, and TYL + MG, hematological parameters were analyzed 24 h post-injection, as presented in Table 2. All measured hematological parameters were maintained within normal physiological ranges; however, neutrophils (NE), eosinophils (EO), and mean corpuscular hemoglobin (MCH) showed significant differences among the groups (*p* < 0.05).

As shown in Table 2, all hematological values in every treatment group remained within the established physiological reference ranges for healthy weaned piglets. Similar to the control, TYL IM injection (Group 2) failed to induce significant changes in NE levels. However, Groups 3 and 4 showed a significant elevation, indicating a potent stimulatory effect of MG on the neutrophil counts (*p* < 0.05), although both values fell within reference ranges. In contrast, EO levels in Group 4 were decreased by 29.7% compared with the control group (0.26 vs. 0.37 × 10^3^/μL, *p* < 0.05; Table 2). Despite these significant differences in NE and EO counts, all measured hematological parameters, including erythrocyte and platelet indices, were within their respective physiological reference intervals. Collectively, TYL and MG administration at the tested doses was well tolerated and was not associated with apparent hematological toxicity.

### 3.3. Plasma Cytokine Profiles

**Figure 3:**
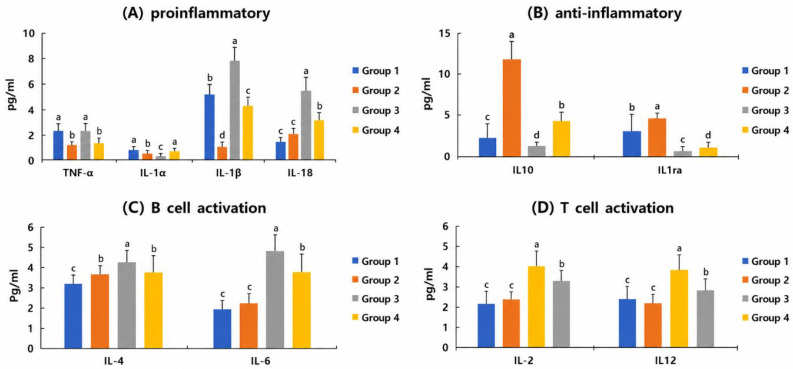
Cytokine profiles reflecting (**A**) pro- and (**B**) anti-inflammatory responses, (**C**) B-cell activation, and (**D**) T-cell activation at 1-day post-dose. Four treatment groups of piglets (*n* = 10 per group) were administered IM injections. Group 1 (saline); Group 2 (TYL, 10 mg/kg); Group 3 (MG, 10 mg/kg); and Group 4 (TYL + MG, 10 mg/kg each). Data are presented as mean ± SD. Bars annotated with different letters (a, b, c, d) differ significantly (*p* < 0.05, one-way ANOVA followed by Tukey’s post hoc test). Abbreviations: TNF-α, tumor necrosis factor-α; IL-10, interleukin-10; IL-1ra, interleukin-1 receptor antagonist; IL-1β, interleukin-1β; IL-4, interleukin-4; IL-6, interleukin-6; IL-2, interleukin-2; IL-12, interleukin-12; IL-18, interleukin-18.

To investigate the immunomodulatory effects of IM injection of TYL alone, MG alone and TYL-MG combination, ten cytokines were analyzed (Figure 3).

IM TYL injection significantly suppressed the levels of TNF-α and IL-1β compared with the control group (Group 1) (*p* < 0.05). In contrast, MG alone (Group 3) significantly elevated the production of IL-1β and IL-18, reaching the highest levels among all groups. When TYL and MG were co-administered (Group 4), TNF-α persisted at reduced levels at a level comparable to Group 2, while IL-1β and IL-18 were maintained at intermediate levels—significantly higher than Group 2 but lower than Group 3 (*p* < 0.05).

Group 2 showed a marked induction of IL-10 and IL-1ra, showing the highest concentrations among all experimental groups (*p* < 0.05). Conversely, Group 3 displayed the lowest levels of both IL-10 and IL-1ra, even lower than those of the control group (Group 1). In the combined treatment group (Group 4), the TYL-induced increase in IL-10 was significantly attenuated to an intermediate level, while IL-1ra remained at a concentration significantly lower than that of Group 1 (*p* < 0.05). The present findings support that TYL administration is associated with a regulatory cytokine response profile.

Activation markers associated with adaptive immunity exhibited a consistent stimulatory response to MG. For B-cell activation markers (IL-4 and IL-6) and T-cell activation markers (IL-2 and IL-12), Group 3 (MG) consistently reached the highest concentrations (*p* < 0.05) compared with all other groups. TYL alone (Group 2) did not significantly affect IL-6, IL-2, or IL-12 levels relative to Group 1 (*p* > 0.05), although it caused a slight increase in IL-4. In the TYL + MG treatment group (Group 4), the stimulatory effects of MG were partially maintained, with IL-4, IL-6, IL-2, and IL-12 levels remaining significantly higher than those in Groups 1 and 2 (*p* < 0.05). These observations indicate a potential immunomodulatory role for MG involving both B-cell- and T-cell-associated cytokine pathways.

### 3.4. Culturable Fecal Microbiota Analysis

**Figure 4:**
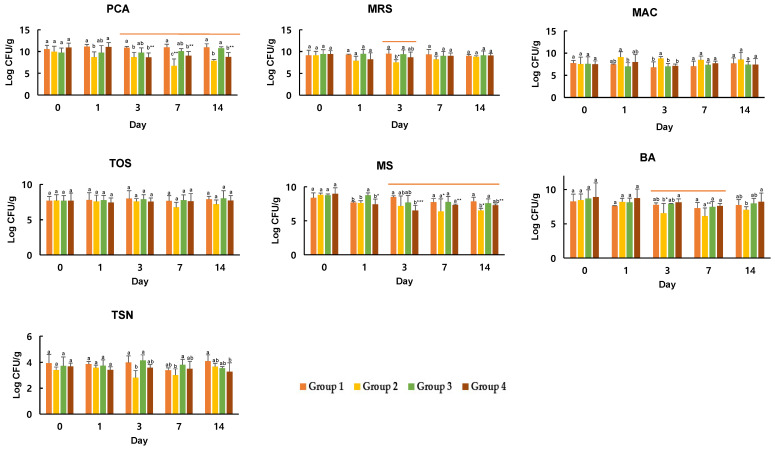
Culture-based bacterial enumeration using selective media following IM administration of TYL, MG and their combination in weaned piglets. Fecal bacterial counts were evaluated using selective and differential culture media on days 0, 1, 3, 7, and 14 after treatment. PCA, total aerobic bacteria; MRS, *Lactobacillus* spp.; MAC, Enterobacteriaceae including *Escherichia coli*; TOS, *Bifidobacterium* spp.; MS, *Staphylococcus* spp.; BA, total hemolytic and non-hemolytic bacteria; TSN, sulfite-reducing anaerobic bacteria including *Clostridium* spp. Four treatment groups were included: Group 1 (saline control), Group 2 (TYL, 10 mg/kg IM), Group 3 (MG, 10 mg/kg IM), and Group 4 (TYL + MG, 10 mg/kg each IM). Data are presented as mean ± SD (*n* = 5). Different lowercase letters (a–c) indicate significant differences among treatment groups at the same time point (*p* < 0.05, one-way ANOVA followed by Tukey’s post hoc test). Asterisks indicate significant differences compared with day 0 within the same group (* *p* < 0.05, ** *p* < 0.01, *** *p* < 0.001).

Culture-based microbial indicators were evaluated using selective culture media at Days 0, 1, 3, 7, and 14 to monitor temporal microbiota-associated changes. As shown in Figure 4, the overall trend revealed decreased bacterial counts in Group 2. In contrast, Groups 3 and 4 exhibited relatively higher counts or recovery trends.

Total aerobic bacteria grown on PCA demonstrated significant group effects (F = 31.085, *p* < 0.001), time effects (F = 5.016, *p* = 0.0012), and group × day interactions (F = 4.346, *p* < 0.001). Group 2 exhibited lower counts, whereas Groups 3 and 4 showed relatively higher counts. At corresponding time points, Group 2 had significantly lower total bacterial counts than Group 1 (*p* < 0.05). However, Groups 3 and 4 had total bacterial counts similar to or higher than those of Group 1.

Culture-based *Lactobacillus* populations were measured using MRS agar. The results indicated that only the group effect was significant (F = 7.366, *p* = 0.0002). As shown in Figure 4, Group 2 exhibited a downward trend in bacterial counts at certain time points, whereas Groups 3 and 4 retained relatively stable counts. Specifically, Group 2 demonstrated a clear decreasing trend compared with the other groups, while Groups 3 and 4 sustained relatively stable bacterial levels. Enterobacteriaceae and *E. coli* populations were assessed using MAC agar. The group effect was significant (F = 7.228, *p* = 0.0002), whereas time and interaction effects were not significant.

The TYL-only group (Group 2) generally exhibited lower bacterial counts, while the MG-only group (Group 3) and the TYL + MG group (Group 4) maintained relatively higher counts. Anaerobic *Bifidobacterium* spp. were measured using TOS agar, with no significant differences observed among groups, over time, or in group × time interactions. Total hemolytic and non-hemolytic bacteria were measured on BA agar. Both group effects (F = 5.981, *p* = 0.0010) and time effects (F = 7.489, *p* < 0.001) were significant, whereas interaction effects were not. This medium supports the growth of various aerobic and anaerobic bacteria. Group 2 displayed a decreasing trend at certain time points, while Groups 3 and 4 tended to maintain relatively higher counts.

Clostridium species, including sulfite-reducing anaerobes such as *C. perfringens*, were assessed using TSN agar. Significant group effects (F = 9.909, *p* < 0.001) and interaction effects (F = 2.330, *p* = 0.013) were observed. Group 2 tended to maintain lower counts, whereas Groups 3 and 4 exhibited relatively higher levels.

In summary, the TYL-alone group exhibited decreased bacterial counts across multiple media, whereas the MG-alone group showed relatively higher culturable bacterial counts. The TYL + MG group exhibited recovery patterns compared with the TYL-only group. This pattern was particularly evident on PCA (total bacteria), MS (Mitis–Salivarius *streptococci*), BA (total bacteria), and TSN (*Clostridium* counts) media. These observations suggest that culture-based fecal microbiota responses varied depending on the treatment group. Notably, the ratio between MRS-grown bacteria (*Lactobacillus*) and MacConkey-grown bacteria (*Enterobacteriaceae*) was used as a culture-based indicator of relative microbial changes. The culture-based Lactobacillus and Enterobacteriaceae populations are considered important indicators of intestinal health following TYL administration. Therefore, changes in these indicators and the culture-based *Lactobacillus*/*Enterobacteriaceae* populations cultured on MAC agar (L/E) ratio after MG administration were analyzed and are presented in Table 3.

As shown in Table 3, on day 0, Groups 3 and 4 exhibited higher L/E ratios than Groups 1 and 2, although the differences were not statistically significant (*p* > 0.05). However, from day 1 onward, the L/E ratio showed clear and statistically significant differences among the groups (*p* < 0.001). The L/E ratios in Group 1 and the MG-alone group (Group 3) were 1.82 and 2.30, respectively. In contrast, the TYL-alone group (Group 2) showed a decrease to −1.22. In the TYL + MG group (Group 4), the L/E ratio increased to 0.67 compared with the TYL-only group. On day 3, Group 2 showed the dominance of *Enterobacteriaceae* over *Lactobacillus* spp., with an L/E ratio of −1.46. Conversely, Group 4 maintained a significantly higher ratio (1.56) than Group 2 (*p* < 0.05). By day 14, the L/E ratios in all treatment groups had returned to positive values.

### 3.5. 16S rRNA Gene Profiling and Alpha Diversity of Fecal Microbiota

**Figure 5:**
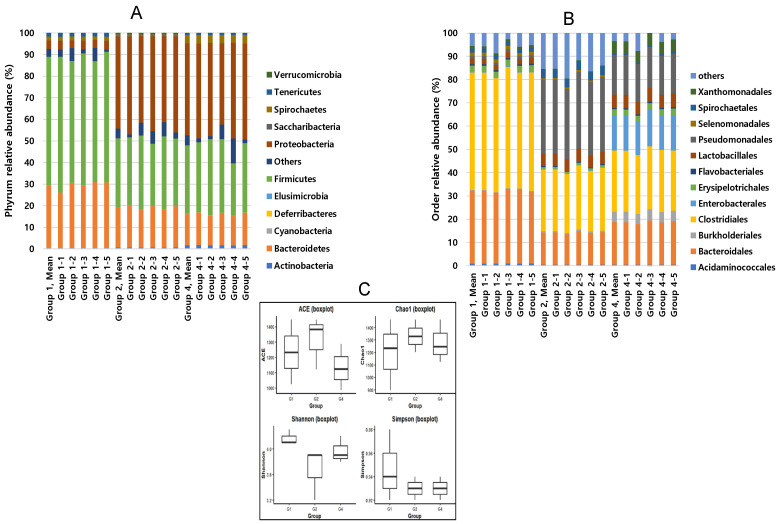
Gut microbiota composition and alpha diversity in weaned piglets after IM administration. (**A**) Relative abundance of bacterial phyla). (**B**) Relative abundance of bacterial orders. (**C**) Alpha diversity indices (ACE, Chao1, Shannon, and Simpson). Boxplots show the median (center line), interquartile range (box), and whiskers representing the data distribution.

Exploratory 16S rRNA gene sequencing was performed on a limited representative subset of fecal samples collected on day 3, selected to characterize microbiota-associated changes following TYL administration, rather than on all experimental animals. At the phylum level, Firmicutes and Bacteroidetes were the dominant phyla in Group 1 (control), accounting for 59.95% and 32.09% of the microbial composition, respectively, whereas Proteobacteria represented a relatively small proportion (3.91%) (Figure 5A). In Group 2 (TYL alone), the relative abundance of Proteobacteria increased to 42.96%, while Firmicutes and Bacteroidetes decreased to 35.92% and 18.81%, respectively. Group 4 (TYL + MG) exhibited altered microbial composition compared with the control and TYL-treated groups, in which Proteobacteria accounted for 42.63% of the total composition, whereas Firmicutes and Bacteroidetes represented 36.02% and 14.56%, respectively. Order-level microbial composition also differed among groups (Figure 5B). Bacteroidales and Clostridiales accounted for a relatively large proportion in Group 1, whereas Enterobacterales, Pseudomonadales, and Xanthomonadales increased in Group 2. Group 4 showed a similar distribution pattern, with minor differences in the relative abundance of bacterial orders. Although the ACE index showed a tendency to vary among groups, no statistically significant differences were detected. Likewise, no significant differences were observed in Chao1, Shannon, or Simpson indices among the experimental groups, indicating that overall microbial richness and diversity were not substantially altered by treatment.

## 4. Discussion

This study investigated whether IM administration of TYL, which is widely used for the treatment of respiratory bacterial infections in weaned piglets, influences immune responses and gut microbiota composition, and whether co-administration of methyl gallate (MG) could attenuate these alterations. Although IM administration is generally considered to exert less direct influence on intestinal microbiota than oral antibiotic treatment, TYL may still affect the intestinal environment through systemic distribution, biliary excretion, and enterohepatic circulation, thereby influencing gut microbial composition [1,3]. Previous studies have suggested that polyphenolic compounds, including MG, possess immunomodulatory and microbiota-regulating properties and may function as immunonutrients [14]. Based on these findings, the present study aimed to determine whether combined IM administration of TYL and MG could mitigate selected immune- and microbiota-associated alterations induced by TYL alone in weaned piglets. Although single IM administration may not fully represent routine clinical treatment practices, this design allowed for the evaluation of early physiological and microbiota-related changes following TYL administration. Because only a single dose was evaluated, these findings should be interpreted as reflecting acute responses under controlled conditions. Therefore, caution is needed when applying these results to repeated treatment protocols or real-world farm conditions.

To evaluate the safety of combined IM administration of TYL + MG, growth performance indicators (ADG, ADFI, and feed efficiency (FE) were analyzed. No significant differences were observed among the treatment groups (*p* = 0.378). These findings indicated that neither TYL nor MG administration significantly affected weight gain under the present experimental conditions. A slightly higher feed intake was observed in Group 4 compared with the other groups; however, no statistically significant differences were detected among the groups (*p* = 0.833). These observations are consistent with previous studies by Urbanová et al. [6] and Vicca et al. [5], which demonstrated that TYL administration during the weaning period does not impair growth performance. Neither MG alone nor in combination with TYL negatively affected growth performance. These results are also consistent with earlier reports describing the physiological safety of polyphenol-based functional compounds [23,24].

Taken together, the present data indicate that MG administration was not associated with adverse effects on growth performance under the conditions of the present study. Because these outcomes were evaluated at the pen level without replicated pens, they should be interpreted as supportive safety information rather than definitive evidence of treatment effects. Consequently, feed intake, growth performance, and feed efficiency outcomes should be regarded as supportive observations within the present experimental design. Although hematological, cytokine, and fecal microbiota analyses were performed using individual animal samples, the absence of replicated pens remains a limitation when interpreting group-level production outcomes. Therefore, future studies employing replicated pen designs are warranted to further validate the present findings.

Next, hematological analyses were conducted to further assess whether MG could serve as a safe functional adjuvant under weaning stress conditions. Because feed intake was measured at the pen level without pen replication and each treatment group was housed in a single pen, pen-related environmental effects could not be completely separated from treatment effects. Therefore, feed intake, growth performance, and microbiota-related findings should be interpreted cautiously and considered supportive observations within the present study design. Although individual animal samples were used for hematological, cytokine, and fecal microbial analyses, the absence of replicated pens should be considered when interpreting group-level outcomes. Future studies with replicated pen designs may help further confirm the present findings.

All hematological parameters remained within normal physiological ranges across all treatment groups. However, within these normal ranges, neutrophil counts exhibited a significant increase in the TYL + MG group (*p* < 0.05), suggesting that MG may influence innate immune responses [15]. Conversely, eosinophil counts showed a decreasing trend, indicating that MG may also play a role in regulating immune-related responses [25,26]. Although statistically significant differences were detected in selected hematological parameters, all hematological values remained within the established physiological reference ranges for healthy weaned piglets. Therefore, these changes were interpreted as transient physiological variations rather than clinically meaningful hematological abnormalities. Therefore, our data may reflect mild acute hematological responses following treatment rather than strong immunomodulatory effects. To further evaluate immunological differences among the treatment groups, plasma cytokine profiles were analyzed. In the TYL-only group, pro-inflammatory cytokines such as TNF-α and IL-1β were suppressed, whereas the regulatory cytokine IL-10 was increased (*p* < 0.05). This pattern aligns with the well-established anti-inflammatory properties of macrolide antibiotics [8]. In contrast, the MG-only group exhibited changes in plasma cytokines associated with adaptive immune responses, which may be related to the regulatory effects of the NF-κB and MAPK signaling pathways [15,27]. Notably, in the TYL + MG group, MG was associated with partial modulation of selected cytokine responses following TYL administration. Because cytokines were measured at a single time point, these findings should be interpreted as reflecting acute immune-associated responses rather than broad immune regulation [17]. Furthermore, the observed increases in IL-6 and IL-10 following MG administration are consistent with the findings of Mechesso et al. [16]. These findings may reflect that MG may influence acute cytokine-associated immune responses following TYL administration. However, because cytokines were measured at a single time point, these findings should be interpreted cautiously.

The impact of IM TYL injection on intestinal bacteria is closely linked to its pharmacokinetic properties. Following IM administration, TYL may be re-exposed to the intestinal tract through biliary excretion and enterohepatic recirculation, thereby exerting antibiotic selection pressure on the gut microbiota [8,9,11]. In the present study, the TYL-only group exhibited an increase in Proteobacteria and a decrease in the culture-based Lactobacillus populations, findings that are consistent with the concept of antibiotic-induced ecological collateral damage [3,11]. Notably, the culture-based *Lactobacillus*/*Enterobacteriaceae* ratio (L/E), a key indicator of intestinal health, was lowered on days 3 and 7 in the TYL-only group, whereas relatively higher ratios were maintained in the TYL + MG group. These findings provide preliminary evidence that MG may alleviate TYL-induced gut dysbiosis under the experimental conditions of the present study [21].

Because microbial measurements were evaluated repeatedly over time, the current results should be interpreted as temporal treatment-associated responses under the conditions of the present study. To further explore these observations, exploratory 16S rRNA gene sequencing was performed on a representative subset of fecal samples collected on day 3, when microbiota-associated changes were most pronounced following TYL administration. Because culture-based selective media do not provide species-level confirmation, the observed bacterial populations should be interpreted as culture-based microbial indicators rather than precise taxonomic measurements. Therefore, on day 3, when variations were most pronounced following TYL exposure, fecal samples were collected for DNA extraction, and 16S rDNA sequencing was performed for microbiota analysis using next-generation sequencing (NGS).

On day 3, fecal samples from the control group (Group 1) predominantly contained Firmicutes and Bacteroidetes. In contrast, fecal samples from the TYL-only group (Group 2) exhibited an increased relative abundance of Proteobacteria, while Firmicutes and Bacteroidetes were reduced. Firmicutes and Bacteroidetes play critical roles in maintaining intestinal metabolic balance and energy homeostasis, whereas the increased relative abundance of Proteobacteria may suggest microbiota-associated alterations following TYL administration. Therefore, the observed increase in Proteobacteria in this study implies that TYL altered microbial community composition. However, because phylum-level compositional data are descriptive in nature, these findings should be interpreted cautiously [3,11]. In the TYL + MG group (Group 4), although the relative abundance of Proteobacteria remained higher than in the control group, the microbial imbalance appeared to be partially alleviated compared with the TYL-only group. Because selective culture media do not provide species-level confirmation, these results should be interpreted as culture-based microbial indicators rather than precise taxonomic measurements. Additional studies with larger sample sizes, inclusion of all treatment groups, and repeated longitudinal sampling will be needed to further confirm these observations.

In our study, ACE and Chao1 indices, reflecting species diversity, tended to increase in the TYL-alone group, although no statistically significant differences were observed. An increase in abundance is usually interpreted as an increase in microbial diversity, but in the context of antibiotic administration, it may also reflect altered microbial community restructuring following antibiotic exposure [11]. This phenomenon confirmed that the distribution of proteobacteria in the TYL-only group was increased. These results suggest that the previously dominant bacterial population was reduced by TYL due to antibiotic selection pressure. These estimates suggest that antibiotic-resistant or resistant environmental bacteria and changes in microbial populations may have occurred. On the other hand, the Shannon index, which reflects microbial diversity and uniformity, decreased in the TYL-alone group. The increase in richness caused by TYL administration reflects the possible disordered shifts in microbial populations due to the opening of ecological niches, and at the same time, the observed reduction in diversity and uniformity changes in microbial community composition due to the abnormal dominance of certain harmful bacteria (Proteobacteria). This finding suggests that the predominance of certain bacterial taxa reduced the overall balance of the gut microbiota. Taken together, these results suggest that MG may play an important role in maintaining the balance and stability of the gut microbiota. Further studies including intestinal inflammatory markers, barrier-function assessment, SCFA analysis, histopathology, and functional microbiota evaluation will help better understand the biological significance of these microbial changes.

Previous studies have reported that MG exerts antimicrobial activity by disturbing bacterial membrane translocation and altering membrane permeability [16,18]. In addition, MG has been confirmed to inhibit the emergence of antibiotic-resistant bacteria and reduce the expression of pathogenic genes [4,17]. These characteristics are consistent with the sustainable antibiotic use strategy proposed by Holman and Chenier [2]. Therefore, the results of this study support our hypothesis that TYL + MG treatment may partially mitigate TYL-induced gut microbiota disruption. The present findings suggest that the co-administration of TYL and MG alleviates the side effects on the gut microbiota and may warrant further investigation for antibiotics. However, the next study should be a pharmacokinetic-based dose and dosing setting study. Previous studies have suggested possible interactions between methyl gallate and drug transport or metabolism pathways. However, because drug concentrations were not measured in the present study, the relevance of these mechanisms to tylosin treatment remains unclear and requires further investigation [28]. Therefore, the combination of TYL and MG may contribute to reducing the primary metabolism of TYL, making systemic drug concentrations more stable [18,27,28]. Future pharmacokinetic studies measuring tylosin and MG concentrations will be necessary to clarify whether such interactions occur under practical treatment conditions. Additional expectations when MG is used as an antibiotic adjuvant are that it can inhibit the emergence of antibiotic-resistant bacteria [1,3]. Second, as reported by Ferreira et al. [14], it was thought that MG could alleviate the physiological disturbances caused by TYL through a biological buffering effect.

Although the present findings do not demonstrate direct enhancement of TYL antibacterial efficacy, they may provide useful information for future studies investigating the supportive role of MG during antimicrobial treatment. Furthermore, because gut microbiota composition can be influenced by environmental conditions, diet, and farm management practices, large-scale validation studies involving piglets raised under diverse farming systems, together with integrated metagenomic and transcriptomic analyses, are necessary to confirm the reproducibility of the present findings and to further elucidate the mechanistic relationship between microbiota alterations and host immune responses following combined TYL and MG treatment. In addition, the relatively small sample size used for 16S rRNA sequencing analysis may limit the generalizability of the microbiota-related findings. Nevertheless, the present study provides preliminary evidence that MG, as a polyphenol-based adjuvant, may mitigate TYL-associated gut microbiota disturbances while maintaining physiological stability in weaned piglets. Considering the growing importance of livestock antimicrobial stewardship, these observations may contribute to the development of adjunctive therapeutic strategies aimed at reducing antibiotic-associated ecological disruption while preserving antimicrobial efficacy.

## 5. Conclusions

In conclusion, the co-administration of MG with TYL via IM injection partially mitigated TYL-induced alterations in plasma cytokine responses and gut microbiota composition in weaned piglets. The recovery of the culture-based *Lactobacillus*/*Enterobacteriaceae* ratio, a key indicator of intestinal microbial balance, occurred more rapidly in the TYL + MG group than in the TYL-alone group. In addition, MG did not completely prevent TYL-associated alterations but was associated with partial attenuation of selected microbiota-associated alterations and partial modulation of selected cytokine responses under the experimental conditions of this exploratory study. Future studies involving repeated dosing, pathogen-challenge models, and larger-scale microbiota profiling are warranted.

## Figures and Tables

**Table 1:** Composition of basal diet (as-fed basis) fed to crossbred weaned piglets.

Ingredients	(%)	Ingredients	(%)	Calculated Composition (%)	
Yellow corn	52.29	Molasses	4.01	^3^ DE (MJ/kg)	14.56
Distillers dried grains	2.01	Lysine, 98%	0.19	^4^ CP	20.47
Soybean oil meal, CP: 48%	10.34	Methionine, 98%	0.10	Crude fat	5.31
Soybean oil meal, CP: 45%	22.63	Choline chloride, 50%	0.05	Isoleucine	0.61
Ground limestone	1.20	Antioxidant	0.02	Lysine	1.25
Dicalcium phosphorous	1.61	Sweetener	0.04	Methionine	0.34
Salt	0.25	^1^ Vitamin premix	0.10	Threonine	0.85
Animal fat	5.02	^2^ Mineral premix	0.10	Valine	0.83
Tryptophan, 98%	0.04			Tryptophan	0.20

^1^ Vitamin premix; vitamin A, 1175 IU; vitamin D3, 1500; vitamin E, 50 IU; vitamin K, 1.75 mg; choline chloride, 750 mg; niacin, 38 mg; calcium pantothenate, 35.75 mg; riboflavin, 10 mg; thiamin, 20 mg; vitamin B12, 27.5 g; biotin, 100 g; and folic acid, 0.5 mg. ^2^ mineral premix; Ca, 0.9%; P, 0.425%; Mg, 0.0125%; Mn (MnO), 35 mg; Fe (FeSO_4_), 152.5 mg; Zn (ZnO), 137.5 mg; Cu (CuSO_4_), 125 mg; and I (CaI_2_O_6_), 0.75 mg. On an as-fed basis, the diet was estimated to contain CF ≈ 3.205%, NDF ≈ 9.141%, ADF ≈ 4.030%, and TDF ≈ 11.335%. Abbreviations: ^3^ DE, digestible energy; ^4^ CP, crude protein.

**Table 2:** Hematological parameters in blood samples collected at 24 h from weaned piglets following IM administration in weaned piglets (mean ± SD; *n* = 10 per group).

Parameters	Group 1	Group 2	Group 3	Group 4
Leukocytes				
WBC (×10^3^/µL, 5.4–25.2)	18.08 ± 1.21	17.91 ± 0.97	18.21 ± 1.32	18.06 ± 1.26
NE (×10^3^/µL, 0.81–13.40)	8.19 ± 0.92 ^a^	8.16 ± 0.83 ^a^	8.84 ± 0.76 ^ab^	8.98 ± 0.65 ^b^
LY (×10^3^/µL, 3.81–14.92)	9.52 ± 0.82	9.63 ± 0.87	9.14 ± 1.92	10.62 ± 0.97
MO (×10^3^/µL, 0.22–1.71)	0.54 ± 0.05	0.54 ± 0.05	0.56 ± 0.04	0.57 ± 0.05
EO (×10^3^/µL, 0.05–0.48)	0.37 ± 0.02 ^a^	0.35 ± 0.01 ^b^	0.31 ± 0.02 ^c^	0.26 ± 0.01 ^d^
BA (×10^3^/µL, 0.01–0.15)	0.03 ± 0.01	0.038 ± 0.01	0.037 ± 0.02	0.027 ± 0.02
Thrombocytes				
PLT (×10^3^/µL, 208–873)	323.8 ± 27.2	331.3 ± 26.2	324.8 ± 26.4	319.3 ± 26.2
MPV (fL, 7.47–16.5)	11.97 ± 1.82	11.44 ± 1.67	12.22 ± 1.87	11.71 ± 1.76
RBC (×10^6^/µL, 5.52–9.11)	8.96 ± 0.93	8.52 ± 1.93	8.84 ± 0.98	8.73 ± 0.99
Hb (g/dL, 8.8–12.7)	13.43 ± 1.21	13.84 ± 1.23	13.05 ± 0.98	13.02 ± 1.34
HCT (%, 28.3–42.7)	44.23 ± 4.76	43.21 ± 6.21	45.23 ± 5.23	45.25 ± 5.76
MCV (fL, 38.4–59.3)	54.22 ± 5.22	54.16 ± 5.43	54.23 ± 6.22	55.11 ± 5.22
MCH (pg, 11.1–18.4)	15.24 ± 1.22 ^a^	17.86 ± 1.76 ^b^	16.44 ± 1.43 ^ab^	16.81 ± 1.23 ^ab^
MCHC(g/dL, 27.9–32.4)	27.14 ± 2.12	27.09 ± 2.26	26.26 ± 1.76	25.24 ± 2.11
RDW(%, 16.4–32.3)	19.63 ± 1.16	19.27 ± 1.26	19.23 ± 1.22	18.22 ± 1.32

Values are presented as mean ± SD. Values with different superscript letters within the same row are significantly different (*p* < 0.05). WBC, white blood cell; NE, neutrophils; LY, lymphocytes; MO, monocytes; EO, eosinophils; BA, basophils; PLT, platelets; MPV, mean platelet volume; RBC, red blood cell count; Hb, hemoglobin concentration; HCT, hematocrit; MCV, mean corpuscular volume; MCH, mean corpuscular hemoglobin; MCHC, mean corpuscular hemoglobin concentration; RDW, red cell distribution width.

**Table 3:** Log-transformed *Lactobacillus*/*Enterobacteriaceae* culture-based *Lactobacillus*/*Enterobacteriaceae* ratio (mean ± SD).

Day	Group 1	Group 2	Group 3	Group 4
0	1.43 ± 0.88 ^a^	1.45 ± 1.30 ^a^	1.87 ± 1.22 ^a^	1.98 ± 0.89 ^a^
1	1.82 ± 0.20 ^a^*	−1.22 ± 1.54 ^c^***	2.30 ± 1.32 ^a^*	0.67 ± 2.05 ^b ns^
3	2.76 ± 1.32 ^a^**	−1.46 ± 0.65 ^c^***	2.09 ± 0.82 ^a^*	1.56 ± 1.31 ^b^*
7	1.91 ± 1.60 ^a ns^	−0.24 ± 1.12 ^b^**	1.65 ± 0.64 ^a ns^	1.35 ± 0.78 ^a ns^
14	1.56 ± 1.25 ^a ns^	0.29 ± 1.75 ^b^*	1.78 ± 0.69 ^a ns^	1.70 ± 1.50 ^a ns^

The culture-based *Lactobacillus*/*Enterobacteriaceae* ratio was calculated based on log10-transformed bacterial counts obtained from MRS (*Lactobacillus* spp.) and MAC (*Enterobacteriaceae*) media and expressed as the difference between log10 values (log10 MRS − log10 MAC). Data are presented as mean ± standard deviation (SD) for each group at each time point. Different superscript letters (a–c) within the same row indicate significant differences among groups (*p* < 0.05). Asterisks indicate significant differences compared with Day 0 within the same group (* *p* < 0.05, ** *p* < 0.01, *** *p* < 0.001; ns, not significant).

## Data Availability

Fecal microbiome analysis was performed using a commercial 16S rRNA sequencing service provided by ChunLab (currently integrated into CJ Bioscience, Seoul, Republic of Korea). As the sequencing was conducted as a commercial analytical service, raw sequencing data were not deposited in a public repository and no accession number is available. The data supporting the findings of this study are included in the article. The data supporting the findings of this study are available from the corresponding author upon reasonable request.

## References

[1] Maron D.F., Smith T.J., Nachman K.E. (2013). Restrictions on antimicrobial use in food animal production: An international regulatory and economic survey. Glob. Health.

[2] Holman D.B., Chénier M.R. (2013). Impact of subtherapeutic administration of tylosin and chlortetracycline on antimicrobial resistance in farrow-to-finish swine. FEMS Microbiol. Ecol..

[3] Kim B.N., Kim H.B., Oh M.D. (2016). Antibiotic control policies in South Korea, 2000–2013. Infect. Chemother..

[4] Yoon Y.K., Kwon K.T., Jeong S.J., Moon C., Kim B., Kiem S., Kim H.S., Heo E., Kim S.W. (2021). Guidelines on implementing antimicrobial stewardship programs in Korea. Infect. Chemother..

[5] Vicca J., Maes D., Jonker L., de Kruif A., Haesebrouck F. (2005). Efficacy of in-feed medication with tylosin for the treatment and control of *Mycoplasma hyopneumoniae* infections. Vet. Rec..

[6] Urbanová Z., Zahradníkova M., Schovánek V., Polák L., Rabas P., Sechser T., Svandová E., Raskova H., Raska K., Janovská D. (1975). Effect of tylosin in pigs. Vet. Med..

[7] Aarestrup F.M., Carstensen B. (1998). Effect of tylosin used as a growth promoter on the occurrence of macrolide-resistant enterococci and staphylococci in pigs. Microb. Drug Resist..

[8] Lee E.B., Lee G.Y., Hossain M.A., Awji E.G., Park S.C. (2024). Gut microbiome perturbation and its correlation with tylosin pharmacokinetics in healthy and infected pigs. Sci. Rep..

[9] Manchester A.C., Webb C.B., Blake A.B., Sarwar F., Lidbury J.A., Steiner J.M., Suchodolski J.S. (2019). Long-term impact of tylosin on fecal microbiota and fecal bile acids of healthy dogs. J. Vet. Intern. Med..

[10] Tang X., Xiong K., Fang R., Li M. (2022). Weaning stress and intestinal health of piglets: A review. Front. Immunol..

[11] Suchodolski J.S., Dowd S.E., Westermarck E., Steiner J.M., Wolcott R.D., Spillmann T., Harmoinen J.A. (2009). The effect of the macrolide antibiotic tylosin on microbial diversity in the canine small intestine as demonstrated by massive parallel 16S rRNA gene sequencing. BMC Microbiol..

[12] Shah S.H., Sheikh I.S., Kakar N., Sumaira, Afzal S., Mehmood K., Rehman H.U. (2022). In vivo analysis of the effect of antibiotic growth promoters, oxytetracycline dihydrate and tylosin phosphate, on intestinal microflora in broiler chickens. Braz. J. Biol..

[13] Yang K., Zhang L., Liao P., Xiao Z., Zhang F., Sindaye D., Xin Z., Tan C., Deng J., Yin Y. (2020). Impact of gallic acid on gut health: Focus on the gut microbiome, immune response, and mechanisms of action. Front. Immunol..

[14] Ferreira C., Vieira P., Sá H., Malva J., Castelo-Branco M., Reis F., Viana S. (2024). Polyphenols: Immunonutrients tipping the balance of immunometabolism in chronic diseases. Front. Immunol..

[15] Liang H., Huang Q., Zou L., Wei P., Lu J., Zhang Y. (2023). Methyl gallate: Review of pharmacological activity. Pharmacol. Res..

[16] Mechesso A.F., Yixian Q., Park S.C. (2019). Methyl gallate and tylosin synergistically reduce membrane integrity and intracellular survival of *Salmonella* Typhimurium. PLoS ONE.

[17] Birhanu B.T., Park N.H., Lee S.J., Hossain M.A., Park S.C. (2018). Inhibition of *Salmonella* Typhimurium adhesion, invasion, and intracellular survival via treatment with methyl gallate alone and in combination with marbofloxacin. Vet. Res..

[18] Choi J.G., Mun S.H., Chahar H.S., Bharaj P., Kang O.H., Kim S.G., Shin D.W., Kwon D.Y. (2014). Methyl gallate from *Galla rhois* successfully controls clinical isolates of Salmonella infection in both in vitro and in vivo systems. PLoS ONE.

[19] Zhou P., Lai J., Li Y., Deng J., Zhao C., Huang Q., Yang F., Yang S., Wu Y., Tang X. (2022). Methyl gallate alleviates acute ulcerative colitis by modulating gut microbiota and inhibiting the TLR4/NF-κB pathway. Int. J. Mol. Sci..

[20] Zhang S., Tang S., Liu Z., Lv H., Cai X., Zhong R., Chen L., Zhang H. (2024). Baicalin restores intestinal damage after early-life antibiotic therapy: The role of the MAPK signaling pathway. Pharmacol. Res..

[21] Cheng B., Huang M., Zhou T., Deng Q., Wassie T., Wu T., Wu X. (2023). Garlic essential oil supplementation modulates colonic microbiota composition and regulates immune response in weaned piglets. Heliyon.

[22] Satora M., Magdziarz M., Rząsa A., Rypuła K., Płoneczka-Janeczko K. (2020). Insight into the intestinal microbiome of farrowing sows following administration of garlic (*Allium sativum*) extract and probiotic bacteria cultures under farming conditions. BMC Vet. Res..

[23] Hong S.G., Sato N., Legrand F., Gadkari M., Makiya M., Stokes K., Howe K.N., Yu S.J., Linde N.S., Clevenger R.R. (2020). Glucocorticoid-induced eosinopenia results from CXCR4-dependent bone marrow migration. Blood.

[24] Khoury P., Stokes K., Gadkari M., Makiya M.A., Legrand F., Hu Z., Klion A., Franco L.M. (2018). Glucocorticoid-induced eosinopenia in humans can be linked to early transcriptional events. Allergy.

[25] Zhang L., Virgous C., Si H. (2019). Synergistic anti-inflammatory effects and mechanisms of combined phytochemicals. J. Nutr. Biochem..

[26] Choi S.J., Shin S.C., Choi J.S. (2011). Effects of myricetin on the bioavailability of doxorubicin for oral drug delivery in rats: Possible role of CYP3A4 and P-glycoprotein inhibition by myricetin. Arch. Pharm. Res..

[27] Li J., Chen C., Yang H., Yang X. (2021). Tea polyphenols regulate gut microbiota dysbiosis induced by antibiotics in mice. Food Res. Int..

[28] He Z., Deng N., Zheng B., Gu Y., Chen J., Li T., Liu R.H., Yuan L., Li W. (2023). Apple peel polyphenol alleviates antibiotic-induced intestinal dysbiosis by modulating tight junction proteins, the TLR4/NF-κB pathway, and intestinal flora. Food Funct..

